# Evaluation of targeted next‐generation sequencing for detection of HPV genotypes and sublineages in cervical liquid‐based cytology SurePath samples from the Danish screening program

**DOI:** 10.1002/ijc.70148

**Published:** 2025-09-11

**Authors:** Karoline Andersen, Jesper Bonde, Marianne Waldstrøm, Maria Vad Jakobsen, Philippe Lamy, Helle Pedersen, Sara Bønløkke, Magnus Stougaard, Torben Steiniche

**Affiliations:** ^1^ Department of Clinical Medicine Aarhus University Denmark; ^2^ Department of Pathology Aarhus University Hospital Denmark; ^3^ Molecular Pathology laboratory, Department of Pathology AHH‐Hvidovre Hospital Denmark; ^4^ Department of Molecular Medicine Aarhus University Hospital Denmark; ^5^ Department of Obstetrics and Gynecology Gødstrup Regional Hospital Denmark; ^6^ Department of Clinical Genetics Aarhus University Hospital Denmark

**Keywords:** cervical cancer screening, HPV, HPV sublineages, liquid‐based cytology, NGS

## Abstract

The carcinogenicity of HPV genotypes is well established. However, HPV genotypes have sublineages with individual risk profiles, and these are much less described with respect to carcinogenicity. Research to characterize HPV sublineages by next‐generation sequencing (NGS) on screening‐derived liquid‐based cytology (LBC) samples is limited because of the technical and quality assurance challenging nature of sublineage analysis. This study aimed to evaluate the feasibility of detecting HPV sublineages from 14 HPV genotypes in SurePath LBC samples from Danish cervical cancer screening. We included 41 HPV plasmids (the Global HPV LabNet DNA Genotyping Proficiency Panel 2023) to quality assure the NGS approach and 120 SurePath LBC samples from the screening program for proof of concept. Our results of the HPV plasmids showed the correct sublineage for all included HPV genotypes except for HPV68b, where the coverage was inadequate for sublineage analysis. The NGS analysis enabled HPV sublineage analysis in 99.1% (112/113) of HPV‐positive SurePath LBC samples. Sublineages belonging to the A lineage were most frequent for HPV16, 18, 31, 33, 35, 39, 51, 52, 58, 59, and 68, while B‐type sublineages showed the highest frequency in HPV45, 56, and 66. The most diverse sublineage data was obtained for HPV31 with sublineages from the A, B, and C lineages. In conclusion, our method enables the identification of HPV sublineages in SurePath LBC screening samples. This information can be used in future studies to determine the usefulness of HPV sublineage analysis in screening settings for risk stratification and clinical management of HPV‐positive women.

## Graphical abstract

What's new?

Human papillomavirus (HPV) assays currently used in cervical cancer screening detect genotypes without providing information on viral lineages and sublineages. However, genotype‐specific sublineages may have distinct risk profiles, which could be harnessed to further improve risk stratification. In this proof‐of‐concept study, a next‐generation sequencing panel and analytical protocol were developed specifically for cervical SurePath liquid‐based cytology samples to identify HPV sublineages. The validated protocol could be applied in future studies to determine the usefulness of HPV sublineages in risk stratification and clinical management.

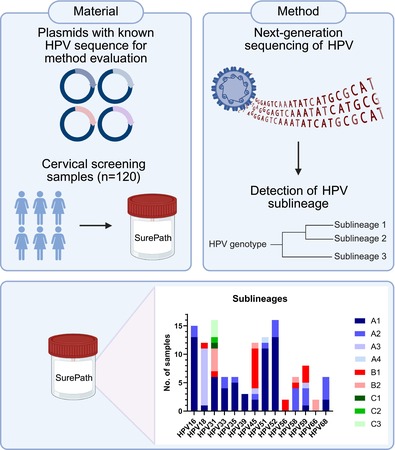

**Abbreviations**
CIN: cervical intraepithelial neoplasia
HPV: human papillomavirus
LBC: liquid‐based cytology
NGS: next‐generation sequencing
SNP: single‐nucleotide polymorphism

## 1 INTRODUCTION

Persistent infection with carcinogenic human papillomavirus (HPV) genotypes is the etiological cause of cervical cancer.^1^, ^2^ This knowledge prompted the development of HPV vaccines for primary prevention of cervical cancer and is also translated into improved cervical cancer screening programs where HPV genotype information is utilized for risk stratification of women.^3^, ^4^ HPV screening for cervical cancer prevention has a high sensitivity for detecting high‐grade cervical intraepithelial neoplasia (CIN) and cancer, as well as a very high negative predictive value.^5^ However, HPV screening has a relatively low specificity, leading to a risk of overtreatment and overmanagement unless effective triage of HPV‐positive screening samples is implemented.^6^, ^7^ HPV genotype detection and characterization can be used as a triage method in this context.^8^, ^9^, ^10^, ^11^, ^12^, ^13^, ^14^

The delineation of HPV genotypes is based on a more than 10% divergence within the virus's L1 gene.^15^ Variations of 1.0%–10% and 0.5%–1.0% across the complete HPV genome further define the division of each HPV genotype into lineages and sublineages, respectively.^16^ Current HPV assays used in cervical cancer screening detect genotypes without providing information on viral lineages and sublineages, and only a few validated tests support extended or full genotyping.^7^

Next‐generation sequencing (NGS) offers in‐depth genetic analysis of HPV. Most studies performing HPV NGS analysis have focused on the implications of viral integration in risk stratification rather than on sublineages, which remain understudied.^17^, ^18^, ^19^, ^20^, ^21^ However, these minor genomic variations may alter the carcinogenic potential of the infection, and sublineages may be operationalized for risk stratification in HPV‐based screening programs. Also, very little evidence exists on sublineage distribution and clinical consequences in screening populations.^18^, ^22^, ^23^, ^24^

This study aimed to establish a targeted NGS panel and analytical protocol designed explicitly for cervical liquid‐based cytology (LBC) samples, enabling comprehensive identification of HPV sublineages across all established carcinogenic HPV genotypes. The developed NGS protocol was first validated using samples containing HPV genomes cloned into plasmid vectors. The validated protocol was applied in analyzing cervical LBC samples from the routine cervical cancer screening program in Denmark to characterize and quantify the distribution of HPV genotype‐specific sublineages.

## 2 MATERIALS AND METHODS

### 2.1 Study materials

This study included 41 HPV genomes cloned into plasmid vectors with known sublineages, provided by the Global HPV LabNet (i.e., the Genotyping Proficiency Panel 2023^25^), and 120 SurePath (Becton Dickinson) cervical LBC samples obtained from women participating in the routine cervical cancer screening program.

#### 2.1.1 HPV plasmid vectors

To assure correct detection of HPV genotype, lineage, and sublineage using our NGS assay, we included 41 HPV genomes cloned into plasmid vectors and diluted in TE buffer containing 10 ng/μL human DNA from the Global HPV LabNet DNA Genotyping Proficiency Panel 2023.^25^ Detection of correct HPV lineage and sublineage was based on the GenBank accession numbers of the specific HPV reference genomes inserted into the plasmid vectors, as provided by the Global HPV LabNet. The HPV reference genomes inserted into plasmid vectors corresponded to the A1 sublineage for all included HPV genotypes, except for HPV35 and HPV68b. For HPV35, the plasmid vector contained an HPV35 prototype reference belonging to the A lineage (sublineage not specified), while the reference for HPV68b belonged to the C1 sublineage. Samples containing the following HPV genotypes, mimicking single‐type infections, were analyzed: 16, 18, 6, 11, 31, 33, 35, 39, 45, 51, 52, 56, 58, 59, 68a, and 68b. Each HPV genotype was included in two samples with different concentrations. HPV16 and 18 were included in concentrations of 1 and 10 international units (IU) per μL, while all other HPV genotypes were included in concentrations of 10 and 100 genome equivalents (GE) per μL. In addition, the study included eight samples with a mix of different HPV genotypes. These samples comprised four distinct mixtures of HPV genotypes: (1) HPV 6, 16, 58, and 68b; (2) HPV 11, 18, 52, and 56; (3) HPV 31, 39, 45, and 68a; and (4) HPV 33, 35, 51, and 59. Each genotype mixture was represented by two samples: the first one contained low concentrations of genotypes, that is, 1 IU/μL for HPV16 and HPV18 and 10 GE/μL for all other HPV genotypes, and the second one contained high concentrations of genotypes, that is, 10 IU/μL for HPV16 and HPV18 and 100 GE/μL for all other HPV genotypes. Lastly, an HPV‐negative control sample was also included.

#### 2.1.2 SurePath cervical liquid‐based cytology samples

Furthermore, we included 120 SurePath cervical LBC samples. The LBC samples were collected as part of the cervical cancer screening program in the Capital Region of Denmark. HPV screening results from the routine BD Onclarity HPV test (Becton Dickinson)^7^, ^11^, ^26^, ^27^, ^28^ were retrieved between April 2024 and June 2024, and samples were selected for inclusion based on the BD Onclarity test results. The BD Onclarity HPV assay contains nine channels for the detection of 14 different HPV genotypes (16, 18, 45, 31, 51, 52, 33/58, 35/39/68, and 56/59/66). For subsequent selection, Ct scores were retrieved from the BD COR platform (Becton Dickinson). Ct values are considered a proxy measure of relative viral load in the samples. Ct values were evaluated to include samples with the broadest possible range of Ct values within the clinical cut‐off of the BD Onclarity HPV assay. We included 108 samples with HPV positivity for one of the nine channels, that is, 12 samples for each of the nine channels. Samples with an HPV‐positive result from one of the three pooled HPV channels (33/58, 35/39/68, and 56/59/66) were subjected to full genotyping using the Allplex HPV HR Detection assay (Seegene). In case of genotype discrepancy between the BD Onclarity and the Allplex assays, the screening standard of care BD Onclarity HPV test result was considered the gold standard. In addition, six HPV‐negative samples and six samples with HPV positivity for multiple infections, as defined by the BD Onclarity HPV test results, were included, resulting in a total of 120 samples.

### 2.2 Sample preparation

#### 2.2.1 HPV plasmid vectors

The HPV plasmid vectors were received from the Global HPV LabNet in a 10 ng/μL human DNA dilution in TE buffer.^25^ No further preparation was performed on these samples prior to library preparation.

#### 2.2.2 SurePath cervical liquid‐based cytology samples

From each sample stored in SurePath vials, 1 mL was transferred to an Eppendorf tube and spun down for 5 min at 14,000 rpm, whereafter the supernatant was removed. The pellet was re‐suspended in a mix of 180 μL phosphate‐buffered saline (10× conc., pH 7.4, Pharmacy product) and 20 μL Proteinase K (Recombinant, PCR grade, Roche Diagnostics). Samples were incubated for 1 h at 56°C (Proteinase K digestion), followed by 1 h at 90°C (reversal of covalent cross‐linking induced by SurePath formaldehyde). DNA was extracted from the processed samples (200 μL) using the BD MAX system (BD Max EXK DNA‐2, Becton Dickinson), yielding 12.5 μL DNA eluate. The eluted DNA was diluted in 37.6 μL Low TE buffer (10 mM Tris, 0.1 mM EDTA, pH 8.0, Thermo Fisher Scientific). DNA concentration was measured on a Qubit Fluorometer using the Qubit 1× dsDNA Quantitation, High Sensitivity Assay Kit (Invitrogen). Samples with a DNA concentration greater than 10 ng/μL were diluted to a concentration of 10 ng/μL using nuclease‐free water.

### 2.3 Next‐generation sequencing panel design

The targeted NGS panel was prepared with the AmpliSeq Designer (Thermo Fisher Scientific) using the same settings described previously for former panel designs.^29^ The panel consists of two pools targeting >96% of 27 HPV genotypes (6, 11, 16, 18, 26, 30, 31, 33, 34, 35, 39, 45, 51, 52, 53, 56, 58, 59, 66, 67, 68, 69, 70, 73, 82, 85, and 97). In addition, each pool contains one primer pair targeting a part of the PPIE and PABPN1 genes, respectively, of the human hg19 genome, functioning as internal controls.

### 2.4 Library and template preparation, chip loading, and sequencing

NGS libraries for each of the two primer pools were prepared from 60 ng of the HPV plasmid vectors from the Global HPV LabNet DNA Genotyping Proficiency Panel 2023^25^ and 4–60 ng of DNA extracted from the LBC samples, depending on the initial DNA concentration. Aside from the DNA input, the sample preparation was identical for all included HPV plasmid vectors and LBC samples. Libraries were prepared using the Ion AmpliSeq Library Kit 2.0 (Thermo Fisher Scientific) following the manufacturer's protocol using half of the reagent volumes stated by the manufacturer for optimized reagent utilization. The samples were subjected to 24 amplification cycles to amplify the targets. IonXpress barcodes (Thermo Fisher Scientific) were added for sample identification. The libraries were quantified using the Ion Library TaqMan Quantitation Kit (Thermo Fisher Scientific), diluted to 40 pM, and pooled. Template preparation and chip loading were performed on the Ion Chef Instrument (Thermo Fisher Scientific) using either the Ion 510 and Ion 520 and Ion 530 Kit‐Chef (Thermo Fisher Scientific) together with the Ion 530 Chip Kit (Thermo Fisher Scientific) or the Ion 540 Kit‐Chef (Thermo Fisher Scientific) together with the Ion 540 Chip Kit (Thermo Fisher Scientific). Sequencing was performed on the Ion GeneStudio S5 Prime System (Thermo Fisher Scientific) using the default coverageAnalysis plugins, except setting the Minimum Aligned Length to 50 base pairs. The sequencing coverage and quality statistics for each sample are summarized in Table S1.

### 2.5 In‐house pipeline for detection of HPV sublineages

**FIGURE 1:**
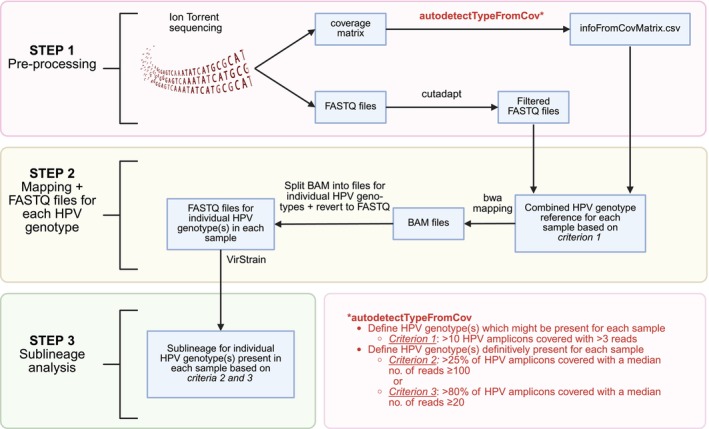
Overview of the bioinformatic workflow.

An overview of the bioinformatic workflow is illustrated in Figure 1. As part of the coverage analysis performed on the Ion GeneStudio S5 Prime System, a coverage matrix containing the read count for each amplicon in all sequenced samples was generated. Based on the data from the coverage matrix, we selected the HPV genotypes that might be present in the samples, setting a cut‐off of >10 amplicons (out of a mean no. of amplicons per HPV genotype of 114) for the same HPV genotype with >3 reads (criterion 1). In addition, two more stringent cut‐offs were applied to determine definitively which HPV genotypes were present in the sample and whether the data were sufficient for sublineage analysis. Either >25% of amplicons should be covered with a median read count ≥100 for a particular HPV genotype (criterion 2) or >80% of amplicons should be covered with a median read count of ≥20 for a particular HPV genotype (criterion 3). Thus, HPV genotyping and sublineage analysis were performed if criteria 2 or 3 were fulfilled. If no HPV genotypes (as selected by criterion 1) fulfilled the requirements for definitive HPV genotyping and sublineage analysis (criteria 2 or 3) in a sample, no further analysis was performed. For samples where data fulfilled the criteria for definitive HPV genotyping and sublineage analysis (criteria 2 or 3), mapping was performed, including alignment to the references for the HPV genotype(s) as selected by criterion 1 and to the reference for the human control. Based on this, a BAM file was generated for each sample and split into several BAM files containing mapped reads for each of the HPV genotypes aligned to. The HPV genotype‐specific BAM files were then reverted to FASTQ files. The FASTQ files were used as input for the definitive HPV genotyping and sublineage analysis if criteria 2 or 3 were met. The sublineage analysis was based on the VirStrain identification tool for viruses.^30^ The HPV sublineage references used for sublineage analysis were retrieved from PaVE^31^ and NCBI. For GenBank accession numbers of all references included in the sublineage analysis, see Table S2.

## 3 RESULTS

### 3.1 HPV plasmid vectors — sublineage assessment

Plasmid vectors containing HPV68a were excluded from sublineage analysis, as the vector provided by the Global HPV LabNet for this genotype included only the L1 gene.

The HPV‐negative control sample from the Global HPV LabNet remained negative when tested using our NGS method. A1 sublineages for HPV16 and HPV18 were correctly identified in samples containing 10 IU/μL. However, for samples with 1 IU/μL, the sequencing depth was insufficient for sublineage determination. For HPV6, HPV11, HPV31, HPV33, HPV39, HPV45, HPV51, HPV52, HPV56, HPV58, and HPV59, the A1 sublineage was correctly identified in samples containing 10 and 100 GE/μL. For the HPV35 plasmid vector containing a prototype A lineage reference, we detected the A1 sublineage in both the 10 and 100 GE/μL samples. For HPV68b, the coverage was inadequate to determine the sublineage, regardless of sample concentration.

### 3.2 SurePath cervical liquid‐based cytology samples — genotyping agreement

A total of 120 SurePath cervical LBC screening samples were included in the study. These comprised six HPV‐negative samples and 114 HPV‐positive screening‐derived samples. In the HPV‐positive group, six samples contained multiple HPV genotypes. One HPV‐screen‐positive sample was excluded prior to analysis due to unsuccessful DNA extraction. All six HPV‐screen‐negative samples remained negative when analyzed with the targeted NGS assay. Complete HPV genotyping concordance between the screening‐determined genotype(s) and the NGS assay was 96.5% (109/113). In 1.8% (2/113) of HPV‐screen‐positive samples, the NGS assay detected an additional HPV genotype not reported by the clinical screening assay: HPV52 and HPV39. In both samples, the HPV genotype identified by BD Onclarity remained the predominant read count genotype in the NGS analysis. In one HPV‐screen‐positive sample (0.9%), no HPV genotypes were detected using the NGS assay, and the sample was excluded from the subsequent sublineage analysis. For this sample, the BD Onclarity assay identified HPV18 with a Ct value of 32.2, which is close to the clinical cut‐off of 34.2, suggesting a low relative viral load of the sample. Finally, in one sample (0.9%), NGS detected HPV16 and HPV68, while BD Onclarity reported HPV45 (Table 1). Co‐infections with non‐carcinogenic HPV genotypes were identified in 12.4% (14/113) of HPV‐positive samples (Table S1).

**TABLE 1:** HPV genotype concordance between the BD Onclarity HPV test and the next‐generation sequencing (NGS) assay.

HPV screening result, BD Onclarity	HPV genotype, Allplex	Concordant with NGS, *n* (%)	Partially concordant with NGS, *n* (%)	Discordant with NGS, *n* (%)	Total no. of samples, *n*
HPV16		11 (100)	‐	‐	11
HPV18		11 (91.7)	‐	1 (8.3)^a^	12
HPV31		12 (100)	‐	‐	12
HPV45		10 (83.3)	1 (8.3)^b^	1 (8.3)^c^	12
HPV51		12 (100)	‐	‐	12
HPV52		11 (91.7)	1 (8.3)^d^	‐	12
P1^e^	HPV33 HPV58	6 (100) 6 (100)	‐ ‐	‐ ‐	12
P2^e^	HPV56 HPV59 HPV66	2 (100) 8 (100) 2 (100)	‐ ‐ ‐	‐ ‐ ‐	12
P3^e^, ^f^	HPV35 HPV39 HPV68	6 (100) 2 (100) 6 (100)	‐ ‐ ‐	‐ ‐ ‐	12^f^
HPV negative		6 (100)			6
*Multiple infections*					
HPV16 + 18		1 (100)	‐	‐	1
HPV16 + 31		1 (100)	‐	‐	1
HPV16 + 52		1 (100)	‐	‐	1
HPV31 + 45 + 51		1 (100)	‐	‐	1
HPV31 + 52		2 (100)	‐	‐	2
Total no. of samples^g^		115 (96.6)	2 (1.7)	2 (1.7)	119^g^

^a^ HPV screening result: HPV18, NGS result: HPV negative.

^b^ HPV screening result: HPV45, NGS result: HPV45 + HPV52.

^c^ HPV screening result: HPV45, NGS result: HPV16 + HPV68.

^d^ HPV screening result: HPV52, NGS result: HPV39 + HPV52.

^e^ P1, P2, and P3 denote the three channels of the BD Onclarity HPV test with pooled HPV genotypes. P1 includes HPV33 and HPV58. P2 includes HPV56, HPV59, and HPV66. P3 includes HPV35, HPV39, and HPV68.

^f^ Two samples contained two different HPV genotypes within P3, that is, 12 samples were positive for HPV genotypes included in the P3 channel.

^g^ Sample no. 120 was excluded prior to analysis due to unsuccessful DNA extraction.

### 3.3 SurePath cervical liquid‐based cytology samples — sublineage assessment

**FIGURE 2:**
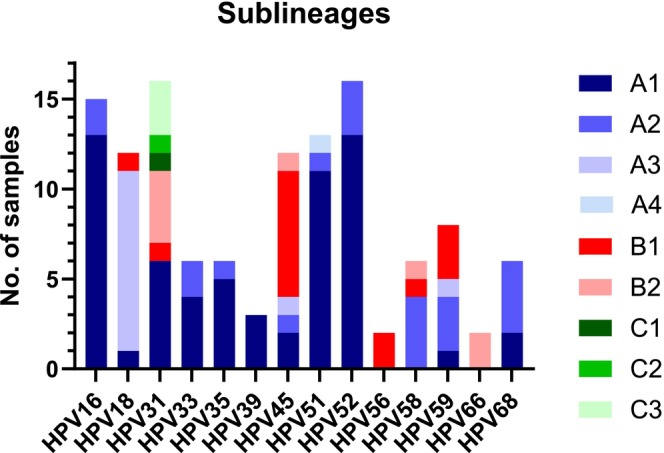
Genotype‐specific HPV sublineages detected in 112 SurePath cervical liquid‐based cytology screening samples.

Of the 113 HPV‐screen‐positive cervical LBC samples, 112 samples were eligible for HPV sublineage analysis. The number of sublineages per HPV genotype ranged from one to six. Sublineages belonging to the genotype‐specific A lineage were the most frequent among samples positive for HPV16, 18, 33, 35, 39, 51, 52, 58, 59, and 68. The B1 sublineage was the most frequently detected in HPV45 and HPV56, while all HPV66‐positive samples were classified as B2 (Table 2, Figure 2). HPV31 presented the highest number of sublineages: six different sublineages from three distinct lineages. The sublineages identified for each HPV genotype are presented in Table 2 and Figure 2. For samples containing multiple HPV genotypes, the identified sublineage is included for all detected HPV genotypes (Table 2 and Figure 2).

**TABLE 2:** Genotype‐specific HPV sublineages detected in 112 SurePath cervical liquid‐based cytology screening samples.

HPV genotype	A1, *n* (%)	A2, *n* (%)	A3, *n* (%)	A4, *n* (%)	B1, *n* (%)	B2, *n* (%)	C1, *n* (%)	C2, *n* (%)	C3, *n* (%)
HPV16	13/15 (86.7)	2/15 (13.3)	‐	‐	‐	‐	‐	‐	‐
HPV18	1/12 (8.3)	‐	10/12 (83.3)	‐	1/12 (8.3)	‐	‐	‐	‐
HPV31	6/16 (37.5)	‐	‐	‐	1/16 (6.3)	4/16 (25.0)	1/16 (6.3)	1/16 (6.3)	3/16 (18.8)
HPV33	4/6 (66.7)	2/6 (33.3)	‐	‐	‐	‐	‐	‐	‐
HPV35	5/6 (83.3)	1/6 (16.7)	‐	‐	‐	‐	‐	‐	‐
HPV39	3/3 (100.0)	‐	‐	‐	‐	‐	‐	‐	‐
HPV45	2/12 (16.7)	1/12 (8.3)	1/12 (8.3)	‐	7/12 (58.3)	1/12 (8.3)	‐	‐	‐
HPV51	11/13 (84.6)	1/13 (7.7)	‐	1/13 (7.7)	‐	‐	‐	‐	‐
HPV52	13/16 (81.3)	3/16 (18.8)	‐	‐	‐	‐	‐	‐	‐
HPV56	‐	‐	‐	‐	2/2 (100.0)	‐	‐	‐	‐
HPV58	‐	4/6 (66.7)	‐	‐	1/6 (16.7)	1/6 (16.7)	‐	‐	‐
HPV59	1/8 (12.5)	3/8 (37.5)	1/8 (12.5)	‐	3/8 (37.5)	‐	‐	‐	‐
HPV66	‐	‐	‐	‐	‐	2/2 (100.0)	‐	‐	‐
HPV68	2/6 (33.3)	4/6 (66.7)	‐	‐	‐	‐	‐	‐	‐

## 4 DISCUSSION

In the present study, we developed and validated a targeted NGS panel and analytical protocol specifically optimized for SurePath cervical LBC screening samples, which enabled comprehensive lineage and sublineage classification of all HPV genotypes classified as carcinogenic to humans. The developed protocol was validated using 41 HPV genomes cloned into plasmid vectors to ensure the quality and reliability of our assay. As proof of concept to evaluate the clinical applicability, we analyzed 120 SurePath cervical LBC samples obtained from women undergoing routine cervical cancer screening in Denmark. The HPV genotype‐specific concordance of HPV‐positive samples was very high (96.5%) between the screening assay (BD Onclarity HPV assay) and the developed NGS assay. Furthermore, sublineage classification was successfully achieved in 112 out of 113 HPV‐positive samples using our in‐house bioinformatics pipeline.

With the exception of HPV68b, which lacked sufficient genome coverage for sublineage analysis, the NGS assay accurately determined the sublineages of all HPV genotypes included in the plasmid vectors. The provided HPV68b genome corresponds to a C1 sublineage reference.^32^ Of note, this was the only non‐A lineage reference included in the plasmids. Since the NGS panel was designed using A1 sublineage references to develop the primer pairs for amplification, this may explain the low genome coverage observed for HPV68b and the consequent inability to determine its sublineage. The inclusion of only the A1 sublineages within the primer pair design of the NGS panel poses a limitation of this method, with the risk of missing the detection of genotype‐specific sublineages that are highly diverse from the A1 sublineage. However, based on the sublineage results from the screening samples, this does not constitute a technical issue. Also, when comparing the diversity of sublineage genomes for different carcinogenic HPV genotypes, HPV68 has the most genetically diverse sublineages. HPV68 sublineage A/B varies from sublineage C/D/E/F by more than 6%, whereas sublineages of other carcinogenic HPV genotypes generally do not differ more than approximately 2% across the complete genomes.^16^, ^32^, ^33^ Hence, the detection problem may be limited to include only HPV68.

For the detection of HPV sublineages, we used an in‐house bioinformatics pipeline based on VirStrain.^30^ This algorithm identifies the most likely HPV sublineage for each genotype present in a sample based on alignment. A limitation of this method is its inability to detect more than one sublineage of a given HPV genotype within the same sample. The occurrence of multiple sublineages has been reported for HPV16.^24^ Also, due to the limited studies on HPV sublineages, the number of identified sublineages varies significantly for different HPV genotypes within the literature. In this study, we retrieved HPV sublineage references from PaVE and NCBI, and therefore, we were only able to re‐identify already known HPV sublineages, without the ability to identify novel HPV sublineages. Accordingly, the number of HPV sublineages identified for each genotype in this study is largely influenced by the extent of prior research, with well‐characterized HPV genotypes typically exhibiting a greater number of recognized sublineages. Even though our method does not enable the identification of novel sublineages, it allows for re‐analysis of our data using the bioinformatics pipeline. Thus, the obtained sequencing data can always be re‐analyzed to include the identification of more sublineages if knowledge of new sublineages emerges.

A strength of this study is the use of NGS for the identification of HPV sublineages, as this technique also enables the identification of other valuable HPV characteristics, such as single‐nucleotide polymorphisms (SNPs) and possible integration of HPV into the host genome. Former studies using NGS for HPV detection have focused primarily on integration and its relation to cancer risk and prognosis.^17^, ^19^, ^20^, ^21^, ^34^, ^35^ A few of these studies have been performed on cervical LBC samples.^20^, ^21^, ^35^ However, to our knowledge, no studies have specified the use of cervical LBC samples collected in SurePath medium, which is used in several cervical cancer screening programs. The presence of formaldehyde in SurePath medium may be challenging when performing extensive molecular analyses.^27^, ^36^ However, our developed NGS assay supports in‐depth HPV analysis directly on SurePath LBC samples, thereby enabling extensive information on HPV infections in cervical cancer screening samples.

The literature is clear that carcinogenicity differs between carcinogenic‐classified HPV genotypes, and this information can be used for risk stratification of HPV‐positive women.^8^, ^9^, ^37^ Currently, a few HPV assays used in cervical cancer screening programs enable extended or full genotyping,^7^ but none provide information regarding the specific lineage or sublineage of the infection. Information on lineages and sublineages of specific HPV genotypes may enhance correct identification of persistent infections and thereby improve the risk stratification of HPV‐positive women, compared to risk stratification based solely on HPV genotypes, where the specificity is suboptimal.^38^ The clinical implication and impact of sublineages on the risk of cancer is small, but a study by Mirabello et al. suggests that women infected with HPV16 A4, D2, and D3 have an increased risk of cervical cancer.^18^ Likewise, Clifford et al. have reported a significant association between infection with the HPV16 A4 and D sublineages and the risk of cervical cancer in a pooled worldwide analysis.^39^ For HPV31, Pinheiro et al. have observed an increased risk of severe cervical dysplasia and cancer when infected with the genotype‐specific sublineages A1, A2, and B2.^40^ This knowledge can be operationalized with the genomic characterization of the woman's HPV infection if confirmed in larger studies. For instance, we found six different HPV31 sublineages belonging to three distinct lineages. Yet, it should be considered that the frequencies of genotype‐specific sublineages depend significantly on the geographical location and included population of studies,^39^, ^40^, ^41^, ^42^ and that the risk of precancer and cancer for specific sublineages may also vary based on the ethnicity/race of the infected woman.^18^, ^40^ Finally, HPV lineages have been suggested to impact the response to radio‐chemotherapy in cervical cancer patients,^43^ offering also a clinical cancer treatment perspective to detailed characterization of HPV genotypes, lineages, and sublineages. Overall, this highlights the importance of HPV sublineage studies within various populations and geographic locations to uncover the full potential of HPV sublineage analysis. The applications of HPV sublineage analysis are wide and may prove valuable in several aspects of HPV‐related diseases, emphasizing the importance of future studies within this area of research.

## 5 CONCLUSION

In conclusion, we developed an NGS panel and analytical protocol to identify HPV genotypes and sublineages in SurePath cervical LBC screening samples. We observed several different genotype‐specific HPV sublineages in this small proof‐of‐concept study. Our validated protocol can be used in future studies to determine the usefulness of HPV sublineage analysis in screening settings for risk stratification and clinical management of HPV‐positive women.

## AUTHOR CONTRIBUTIONS

**Karoline Andersen:** Writing – original draft; funding acquisition; conceptualization; investigation; methodology; validation; visualization; formal analysis; project administration; data curation. **Jesper Bonde:** Conceptualization; writing – review and editing; supervision; resources. **Marianne Waldstrøm:** Conceptualization; writing – review and editing; supervision. **Maria Vad Jakobsen:** Conceptualization; writing – review and editing; supervision. **Philippe Lamy:** Writing – review and editing; software; data curation; formal analysis. **Helle Pedersen:** Resources; writing – review and editing. **Sara Bønløkke:** Methodology; writing – review and editing; supervision. **Magnus Stougaard:** Conceptualization; funding acquisition; writing – review and editing; methodology. **Torben Steiniche:** Conceptualization; writing – review and editing; project administration; supervision; funding acquisition.

## FUNDING INFORMATION

This research was funded by Sygeforsikringen “danmark” (grant no. 2022‐0259) and Fabrikant Einar Willumsens Mindelegat.

## CONFLICT OF INTEREST STATEMENT

J.B. declares test kits and study co‐funding from BD Diagnostics, Seegene, and National Cancer Institute (Brazil) to the institution, and honorariums from BD Diagnostics, Copan S.p.a., Seegene, and British Columbia Cancer Screening Programs (CAN). All other authors declare no conflicts of interest.

## ETHICS STATEMENT

The present study was conducted in accordance with the Declaration of Helsinki and approved by the local Ethical Committee of the Central Denmark Region [jr. no. 1‐10‐72‐74‐22], who waived the requirement for written informed consent for the specific sequencing analyses performed in this study. The study was registered in the Central Denmark Region's Internal Database on Research Projects [1‐16–‐02‐164‐23]. A GDPR data handling agreement approval was established (p‐2024‐17364) between the Central Denmark Region and the Capital Region of Denmark.

## Supporting information


**APPENDIX S1:** Supporting information.

**TABLE S1:** Summary of sequencing coverage and quality statistics for HPV sequencing.
**TABLE S2:** GenBank accession numbers for sublineage analysis.

## Data Availability

Derived data supporting this study's findings are available within the article and its supplementary materials. Other data that support the findings of this study are available from the corresponding author upon request.
