# Supplementary material for: Evaluation of targeted next‐generation sequencing for detection of HPV genotypes and sublineages in cervical liquid‐based cytology SurePath samples from the Danish screening program
Source: Int J Cancer. 2025 Sep 11;158(1):193–201. doi: 10.1002/ijc.70148 (PMC12588552; doi:10.1002/ijc.70148)
Supplement: Supplementary file 1 — APPENDIX S1: Supporting information. [file IJC-158-193-s001.pdf]

Evaluation of Targeted Next-generation Sequencing for Detection of HPV Genotypes and Sublineages in Cervical Liquid-based Cytology SurePath Samples from the Danish Screening Program

Karoline Andersen, Jesper Bonde, Marianne Waldstrøm, Maria Vad Jakobsen, Philippe Lamy, Helle Pedersen, Sara Bønløkke, Magnus Stougaard, Torben Steiniche.

Table of contents

Table S1. Summary of sequencing coverage and quality statistics for HPV sequencing is available as a separate Excel file.

Table S2. GenBank accession numbers for sublineage analysis is available as a separate Excel file.
